# A study on the correlation between novel inflammatory indices and severity of coronary artery lesions in patients with stable coronary artery disease

**DOI:** 10.3389/fcvm.2026.1760308

**Published:** 2026-06-04

**Authors:** Ying Li, Zinan Zhao, Hongxi Zhang, Botao Sang, Sainan Li, Dachuan Guo, Xiaohan Zhao, Xiangfei Liu, Xiaoshuo Li, Deping Liu

**Affiliations:** 1 Peking University Fifth School of Clinical Medicine, Beijing, China; 2 Department of Cardiology, Beijing Hospital, National Center of Gerontology, Institute of Geriatric Medicine, Chinese Academy of Medical Sciences, Beijing, China; 3 Department of Pharmacy, Beijing Hospital, National Center of Gerontology, Institute of Geriatric Medicine, Chinese Academy of Medical Sciences, Beijing, China; 4 Health Service Department of the Guard Bureau of the Joint Staff Department, Beijing, China; 5 Medical School, University of Chinese Academy of Sciences, Beijing, China; 6 Graduate School, Chinese Academy of Medical Sciences and Peking Union Medical College, Beijing, China; 7 The Key Laboratory of Geriatrics, Beijing Institute of Geriatrics, Beijing Hospital, National Center of Gerontology, National Health Commission, Institute of Geriatric Medicine, Chinese Academy of Medical Sciences, Beijing, China

**Keywords:** coronary artery, correlation, Gensini score, novel inflammatory indices, stable coronary artery disease

## Abstract

**Background and Objectives:**

The novel inflammatory indices integrate information from multiple immune cells, but their application in stable coronary artery disease (SCAD) remains limited. This research aims to explore the association between the novel inflammatory indices and the degree of coronary artery lesions in SCAD patients.

**Methods:**

This retrospective analysis involved 2,424 patients of SCAD who received coronary angiography at Beijing Hospital from January 2021 to January 2024. Participants were assigned to groups with mild and severe coronary artery lesions according to the Gensini score. Based on whole blood cell parameters, we calculated the neutrophil-lymphocyte ratio (NLR), monocyte-lymphocyte ratio (MLR), platelet-lymphocyte ratio (PLR), systemic immune inflammation index (SII), systemic inflammatory response index (SIRI), and systemic immune inflammatory response index (SIIRI) and analyzed their links to the degree of coronary artery lesions.

**Results:**

All novel inflammatory indices exhibited positive associations with the Gensini score (*P* < 0.05), among which SIRI (*r* = 0.156, *P* < 0.001) and NLR (*r* = 0.154, *P* < 0.001) showed the strongest correlations. Multivariate logistic regression analysis demonstrated that MLR (OR = 4.19, 95% CI: 1.46–11.98, *P* = 0.008), NLR (OR = 1.20, 95% CI: 1.09–1.32, *P* < 0.001), and SIRI (OR = 1.31, 95% CI: 1.11–1.54, *P* = 0.001) were independent predictors of severe coronary artery lesions.

**Conclusion:**

NLR, MLR, and SIRI demonstrate notable associations with the extent of coronary artery lesions in individuals with SCAD and can be used as non-invasive risk assessment indicators. This research provides novel perspectives for the study of inflammatory mechanisms and risk assessment of SCAD patients.

## 1 Introduction

Globally, coronary atherosclerotic heart disease (CAD) has emerged as a key public health concern threatening human well-being. Stable coronary artery disease (SCAD), a common type of CAD, is characterized by the progressive development of atherosclerotic plaques. Although clinical manifestations are relatively stable, the chronic inflammatory response of plaques continues to drive the progression of coronary artery lesions. Accurate early assessment of lesion severity is key to optimizing clinical management. Inflammation occupies a pivotal position in the occurrence and development of CAD, spanning the entire course from the onset of atherosclerosis to plaque rupture (1). Traditional inflammatory indicators like white blood cell (WBC) count (2) and C-reactive protein (CRP) (3), although widely used, are limited in their ability to reflect the complex immune-inflammatory network comprehensively.

Recently, novel inflammatory indices have become a research focus because of their capability to integrate proportions or counts of multiple immune cells. Examples include single inflammatory indices like the monocyte-lymphocyte ratio (MLR), neutrophil-lymphocyte ratio (NLR), and platelet-lymphocyte ratio (PLR), as well as composite indices such as the systemic immune-inflammatory index (SII), systemic inflammatory response index (SIRI), and systemic immune-inflammatory response index (SIIRI). Such indicators provide a more comprehensive evaluation of the body's inflammatory condition through the integration of multiple cell types. Numerous studies have validated the clinical significance of novel inflammatory markers in coronary artery disease. Elevated NLR and MLR were related to an increasing risk of acute coronary syndrome (ACS) (4) and the severity of coronary artery lesions and poor prognosis of ACS patients (5, 6). PLR was an independent prognostic indicator for major adverse cardiovascular events among individuals suffering from acute myocardial infarction (AMI) (5, 7). SII was found to predict lesion complexity and adverse events in ACS patients (8, 9). SIRI was associated with the increase of cardiovascular event risk, particularly in patients experiencing metabolic abnormalities such as diabetes (10, 11). Elevated SIIRI levels were related to poor cardiovascular outcomes in CAD patients, particularly those with AMI, and could act as a valuable marker to recognize patients at high risk following percutaneous coronary intervention (PCI) (12, 13).

The SYNTAX score focuses on complex lesions (such as bifurcations and calcifications) and severe lesions (such as left main lesions), which is primarily used to guide revascularization strategies rather than simply assessing lesion severity (14, 15). The Gensini score is applied to quantify the severity and scope of vascular lesions in CAD patients. Compared with the SYNTAX score, a key advantage of the Gensini score is its ability to reflect early changes and mild stenosis in atherosclerotic disease (16).

Although novel inflammatory markers have demonstrated potential applications in the assessment of risk and prognosis in CAD, research in SCAD remains limited. Current studies have primarily focused on patients with ACS and typically concentrated on single markers, lacking comparative analyses across different markers. This study aims to identify the relationship between novel inflammatory indices (NLR, MLR, PLR, SII, SIRI, and SIIRI) and the degree of coronary artery lesions in SCAD patients. We hope this will demonstrate their clinical value as non-invasive biomarkers and promote their standardized application in the management of coronary heart disease, thereby providing new insights into early risk stratification and individualized treatments in SCAD.

## 2 Materials and methods

### 2.1 Participants

**Figure 1:**
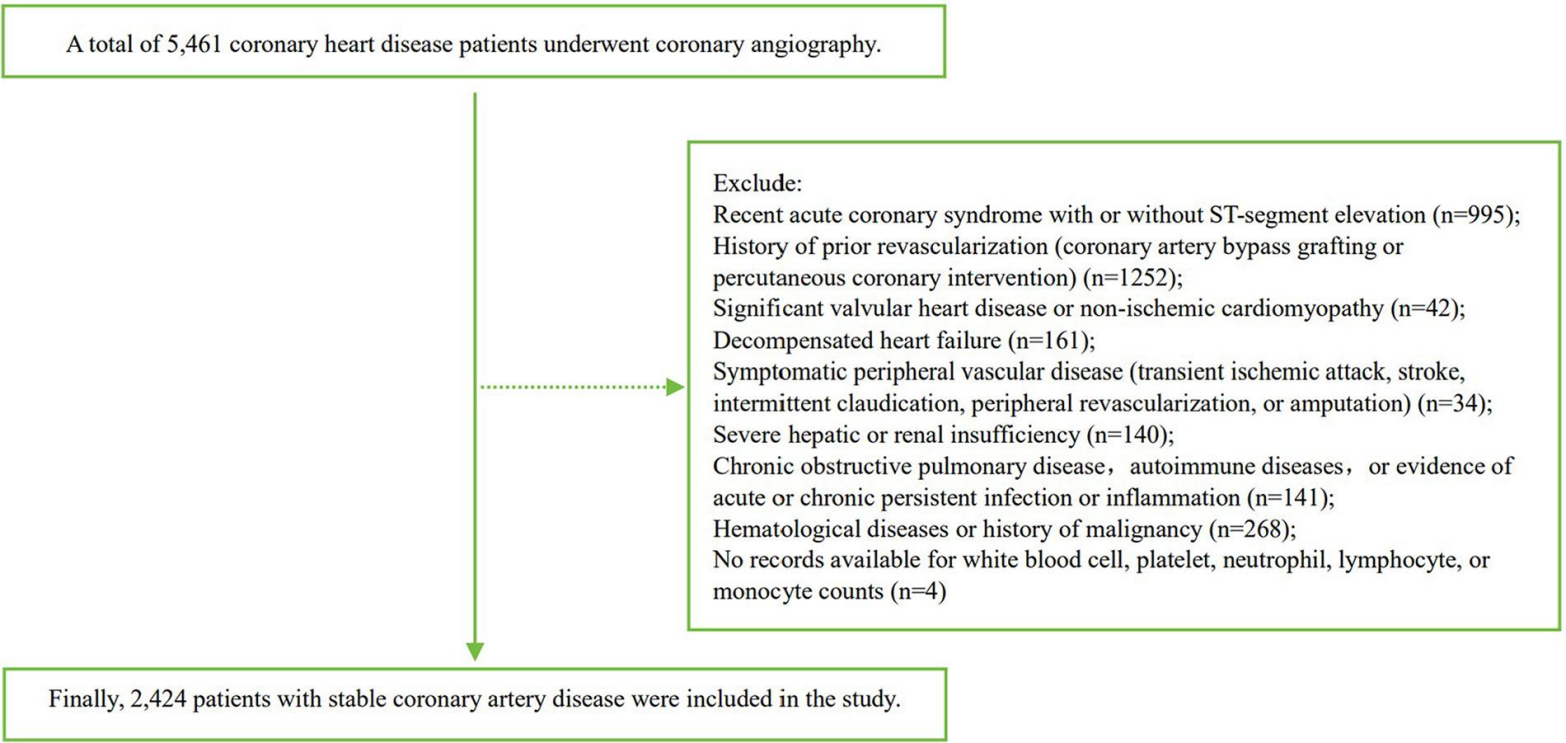
The participants of this study.

We enrolled patients admitted to Beijing Hospital for SCAD and received coronary angiography (CAG) from January 2021 to January 2024. The recommendation for CAG depended on either a positive result of non-invasive load tests or a clinical presumption of severe coronary artery stenosis. Among a total of 5,461 patients who underwent CAG and met the CAD diagnostic criteria, 3,037 were excluded, and 2,424 SCAD patients were ultimately included (Figure 1). This research conforms to the guidelines specified in the Declaration of Helsinki and has obtained approval from the Ethics Committee of Beijing Hospital (Ethics Number: 2025BJYYEC-KY167-01).

### 2.2 Coronary angiography

Two doctors reviewed the CAG images that included at least two orthogonal views to judge the degree and location of coronary lesions and calculated the Gensini score. The scoring criteria were outlined as follows: stenosis ≤25% scored 1 point, stenosis 26%–50% scored 2 points, stenosis 51%–75% scored 4 points, stenosis 76%–90% scored 8 points, stenosis 91%–99% scored 16 points, and complete occlusion scored 32 points. The left main artery (LM) coefficient was 5, the proximal segment coefficient of the left anterior descending artery (LAD) was 2.5, the middle segment coefficient of the LAD was 1.5, the distal segment coefficient of the LAD was 1.0, the proximal segment coefficient of the left circumflex artery (LCX) was 2.5, and the middle and distal segment coefficients of the LCX were 1.0. The coefficients for the proximal, middle, and distant sections of the right coronary artery (RCA) were all 1.0, and the coefficients for other small branch segments were 0.5 (17). Patients were categorized into the mild coronary artery lesions group (Gensini score 1–20) and the severe coronary artery lesions group (Gensini score > 20) (18, 19). SCAD was diagnosed based on the current guideline recommendation (20).

### 2.3 Data collections

On the day of admission, basic demographic characteristics [gender, age, body mass index (BMI), smoking and drinking conditions], medical history (diabetes, hypertension, dyslipidemia, etc.), and NHYA classification of all participants were recorded. Before coronary angiography, we collected the initial fasting venous blood test results from the electronic medical record system after the patient's admission: blood cell count [including WBC, neutrophils, monocytes, lymphocytes, platelets, hemoglobin (HGB), red blood cells (RBC)], glycated haemoglobin (HbA_1_c), total cholesterol (TC), triglycerides (TG), low-density lipoprotein cholesterol (LDL-C), high-density lipoprotein cholesterol (HDL-C), alanine transaminase (ALT), aspartate transaminase (AST), creatinine (Scr), estimated glomerular filtration rate (eGFR), urea nitrogen (Urea), uric acid (UA), D-Dimer, B-type natriuretic peptide (BNP), etc. In addition, cardiac echocardiography data within one month before CAG were collected to obtain information on left ventricular ejection fraction (LVEF).

### 2.4 Calculating new inflammatory indices based on blood cell counts

The formulas for each new inflammatory index were as follows: NLR = neutrophil/lymphocyte; MLR = monocyte/lymphocyte; PLR = platelet/lymphocyte; SII = platelet × neutrophil/lymphocyte; SIRI = neutrophil × monocyte/lymphocyte; SIIRI = neutrophil × monocyte × platelet/lymphocyte.

### 2.5 Statistical analysis

Statistical analysis was carried out by SPSS 17.0 and R 4.4.1. The Shapiro–Wilk test was applied to evaluate the normality of all continuous variables. For descriptive statistics, we reported normally distributed continuous variables as the mean ± standard deviation, skewed continuous variables as the median and interquartile range, and categorical variables as the number and frequency percentage. Depending on the needs, we adopted the Kruskal–Wallis test, variance analysis, or chi-square test to compare differences in continuous or categorical variables across different groups. Correlation analysis was performed by calculating Spearman's coefficient to identify the correlations between different novel inflammatory indices and the Gensini score and other variables. Multivariate logistic regression analysis was performed concerning all potential confounding factors. In addition, we graphed the receiver operating characteristic (ROC) curve to test the discriminatory ability of different novel inflammatory indices for coronary artery lesion severity. We applied restricted cubic splines (RCS) to analyze the dose-response relationship between novel inflammatory indices and coronary artery lesion severity. Two-sided *P* < 0.05 was regarded as statistically significant.

## 3 Results

### 3.1 Baseline characteristics of participants

The baseline clinical features and laboratory parameters of the participants are shown in Tables 1, 2. Overall, 1,129 individuals were assigned to the severe coronary artery lesion group (study group), and 1,295 patients were assigned to the mild coronary artery lesion group (control group). The percentage of males in the severe coronary artery lesions group was 73.87%, notably higher than that in the mild coronary artery lesions group (61.78%). With regard to lifestyle and comorbidities, no meaningful differences were found between the two groups in traditional risk factors, including BMI, diabetes, hypertension, and smoking history (*P* > 0.05). In terms of indicators related to metabolism and cardiac function, the severe coronary artery disease group showed significantly elevated levels of HbA_1_c, Scr, and TG, as well as reduced levels of HDL-C (*P* < 0.05). BNP markedly increased in the severe coronary artery lesions group (34.08 vs. 27.51 pg/mL, *P* < 0.001).

**Table 1:** The baseline clinical features of the participants.

Variables	Total (*n* = 2,424)	Mild coronary artery lesion (*n* = 1,295)	Severe coronary artery lesion (*n* = 1,129)	*P*-value
Age (years)	65.00 (58.00, 70.00)	65.00 (59.00, 70.00)	65.00 (58.00, 69.00)	0.021
Male, n(%)	1,634 (67.41)	800 (61.78)	834 (73.87)	<0.001
BMI (kg/m^2^)	25.71 (23.67, 27.92)	25.69 (23.73, 27.91)	25.71 (23.61, 27.99)	0.932
Hypertension, n(%)	1,800 (74.26)	956 (73.82)	844 (74.76)	0.600
Diabetes, n(%)	1,091 (45.01)	571 (44.09)	520 (46.06)	0.332
Smoker, n(%)	1,123 (46.33)	609 (47.03)	514 (45.53)	0.460
Drinker, n(%)	1,011 (41.71)	545 (42.08)	466 (41.28)	0.687
NYHA level, n(%)				0.210
I∼II	2,230 (92.00)	1,183 (91.35)	1,047 (92.74)	
III∼IV	194 (8.00)	112 (8.65)	82 (7.26)	
LVEF, M (Q₁, Q₃)	0.64 (0.60, 0.65)	0.65 (0.60, 0.66)	0.63 (0.60, 0.65)	<0.001
LM, n(%)	146 (6.02)	2 (0.15)	144 (12.75)	<0.001
Number of vessels, n(%)				<0.001
≤1	860 (35.48)	713 (55.06)	147 (13.02)	
≥2	1,564 (64.52)	582 (44.94)	982 (86.98)	

**Table 2:** The baseline laboratory parameters of the participants.

Variables	Total (*n* = 2,424)	Mild coronary artery lesion (*n* = 1,295)	Severe coronary artery lesion (*n* = 1,129)	*P*-value
WBC (×10^9^/L)	6.00 (5.06, 7.06)	5.78 (4.92, 6.87)	6.22 (5.24, 7.25)	<0.001
Lymphocyte (×10^9^/L)	1.82 (1.46, 2.20)	1.83 (1.47, 2.20)	1.80 (1.45, 2.21)	0.605
Neutrophil (×10^9^/L)	3.50 (2.80, 4.35)	3.35 (2.68, 4.15)	3.71 (2.92, 4.35)	<0.001
Monocyte (×10^9^/L)	0.41 (0.34, 0.50)	0.40 (0.33, 0.49)	0.42 (0.35, 0.51)	0.477
HGB (g/dL)	13.70 (12.70, 14.60)	13.60 (12.60, 14.60)	13.70 (12.70, 14.60)	0.073
RBC (×10^12^/L)	4.43 ± 0.48	4.41 ± 0.48	4.46 ± 0.48	0.005
Platelet (×10^9^/L)	205.00 (173.00, 242.00)	204.00 (173.00, 241.00)	205.00 (174.00, 242.00)	0.550
HbA1c (%)	6.20 (5.80, 7.00)	6.10 (5.70, 6.80)	6.30 (5.90, 7.20)	<0.001
Urea (mmol/L)	5.29 (4.48, 6.26)	5.34 (4.50, 6.28)	5.22 (4.45, 6.21)	0.185
UA (mg/dL)	5.77 (4.83, 6.78)	5.70 (4.77, 6.70)	5.83 (4.91, 6.86)	0.049
Scr (µmol/L)	71.00 (61.00, 81.00)	70.00 (59.00, 80.00)	72.00 (62.00, 82.00)	0.001
eGFR (mL/min/1.73m^2^)	91.43 (82.99, 97.84)	91.26 (82.74, 97.70)	91.70 (83.05, 98.02)	0.435
AST (U/L)	17.00 (15.00, 21.00)	18.00 (15.00, 21.00)	17.00 (15.00, 21.00)	0.393
ALT (U/L)	18.00 (13.00, 25.00)	18.00 (13.00, 24.00)	18.00 (13.00, 25.00)	0.422
TC (mmol/L)	3.79 (3.22, 4.48)	3.81 (3.27, 4.48)	3.75 (3.18, 4.47)	0.152
TG (mmol/L)	1.27 (0.93, 1.79)	1.24 (0.91, 1.73)	1.30 (0.94, 1.84)	0.030
LDL-C (mmol/L)	2.17 (1.71, 2.76)	2.18 (1.73, 2.74)	2.16 (1.70, 2.79)	0.923
HDL-C (mmol/L)	1.05 (0.91, 1.24)	1.08 (0.93, 1.29)	1.03 (0.88, 1.20)	<0.001
BNP (pg/mL)	30.23 (15.18, 61.72)	27.51 (14.05, 56.05)	34.08 (16.14, 68.80)	<0.001
ALB (g/L)	40.00 (38.00, 42.00)	40.00 (38.00, 42.00)	40.00 (38.00, 42.00)	0.089
NLR	1.89 (1.46, 2.55)	1.81 (1.40, 2.46)	2.00 (1.53, 2.69)	<0.001
MLR	0.23 (0.18, 0.29)	0.22 (0.18, 0.28)	0.23 (0.18, 0.30)	<0.001
PLR	112.43 (89.92, 142.53)	111.27 (89.06, 140.23)	114.42 (91.47, 145.26)	0.155
SII	389.41 (278.03, 544.92)	370.46 (264.47, 513.03)	410.56 (303.78, 571.88)	<0.001
SIRI	0.79 (0.55, 1.16)	0.73 (0.52, 1.06)	0.85 (0.59, 1.25)	<0.001
SIIRI	158.96 (104.25, 247.86)	147.08 (96.46, 228.99)	171.57 (114.14, 266.50)	<0.001

Substantial variations in inflammatory indicators were observed between the two groups. The WBC, neutrophil, and monocyte counts were elevated in the severe coronary artery disease group (*P* < 0.001), while there were no significant differences in lymphocyte counts between the two groups (*P* = 0.605). Among the novel inflammatory indices, the severe coronary artery disease group had significantly higher NLR (2.00 vs. 1.81, *P* < 0.001), MLR (0.23 vs. 0.22, *P* < 0.001), SII (410.56 vs. 370.46, *P* < 0.001), SIRI (0.85 vs. 0.73, *P* < 0.001), and SIIRI (171.57 vs. 147.08, *P* < 0.001). In comparison, PLR exhibited no notable difference between the different groups (*P* = 0.155).

### 3.2 Association between novel inflammatory indices and other parameters

To investigate the relationship between various novel inflammatory indices and the Gensini score, as well as other clinical parameters, Spearman correlation coefficients were computed (Table 3). All novel inflammatory indices showed a significant but weak positive correlation with the Gensini score (*P* < 0.05), indicating that a higher inflammatory level corresponds to a more severe degree of coronary artery lesions. Among these, SIRI (*r* = 0.156, *P* < 0.001) and NLR (*r* = 0.154, *P* < 0.001) exhibited the strongest correlation with the Gensini score. In addition, MLR indicated a positive association with age (*r* = 0.140, *P* < 0.001), implying that age may be associated with elevated MLR. NLR (*r* = 0.058, *P* = 0.012) and PLR (*r* = 0.051, *P* = 0.028) showed weak positive correlations with age, while composite indices (SII, SIRI, SIIRI) showed no significant relationship with age. NLR, MLR, and SIRI were negatively correlated with LVEF (*P* < 0.05), indicating that inflammation may be involved in the pathological process of cardiac function deterioration. In addition, HDL-C was negatively correlated with all inflammatory indices except PLR (*P* < 0.05). Notably, the overall correlation coefficient *r* was relatively low, suggesting that inflammation is merely one important contributing factor to the progression of coronary artery lesions. Clinical evaluation should therefore be based on a comprehensive assessment of multiple associated factors.

**Table 3:** Association between novel inflammatory indices and other parameters.

Variable	NLR		MLR		PLR		SIRI		SII		SIIRI	
	*r*	*P*	*r*	*P*	*r*	*P*	*r*	*P*	*r*	*P*	*r*	*P*
Gensini	0.154	<0.001	0.097	<0.001	0.056	0.016	0.156	<0.001	0.120	<0.001	0.137	<0.001
Age	0.058	0.012	0.140	<0.001	0.051	0.028	0.024	0.292	−0.034	0.112	−0.038	0.102
BMI	0.013	0.564	−0.007	0.730	−0.071	0.002	0.047	0.043	0.011	0.621	0.043	0.065
LVEF	−0.074	0.001	−0.080	<0.001	−0.046	0.046	−0.080	0.001	−0.040	0.064	−0.055	0.017
WBC	0.295	<0.001	0.070	<0.001	−0.197	<0.001	0.563	<0.001	0.436	<0.001	0.628	<0.001
HGB	0.091	<0.001	0.072	0.002	−0.132	<0.001	0.221	<0.001	0.069	0.004	0.164	<0.001
RBC	0.099	<0.001	0.054	0.009	−0.086	<0.001	0.201	<0.001	0.119	<0.001	0.190	<0.001
HbA1c	0.021	0.341	−0.014	0.521	−0.065	0.007	0.077	0.001	0.039	0.104	0.084	<0.001
Urea	0.050	0.026	0.075	<0.001	−0.060	0.015	0.073	0.001	−0.014	0.700	0.018	0.393
UA	0.044	0.048	0.042	0.040	−0.091	<0.001	0.104	<0.001	0.008	0.700	0.077	0.001
Scr	0.137	<0.001	0.177	<0.001	−0.054	0.028	0.209	<0.001	0.062	0.010	0.143	<0.001
eGFR	−0.083	<0.001	−0.121	<0.001	0.000	0.100	−0.093	<0.001	−0.011	0.608	−0.040	0.080
AST	−0.070	0.003	−0.003	0.905	−0.094	<0.001	−0.041	0.072	−0.104	<0.001	−0.065	0.004
ALT	−0.037	0.109	−0.022	0.311	−0.094	<0.001	0.019	0.410	−0.030	0.169	0.018	0.430
TC	−0.150	<0.001	−0.147	<0.001	−0.004	0.886	−0.128	<0.001	−0.025	0.244	−0.043	0.596
TG	−0.020	0.345	−0.114	<0.001	−0.068	0.00	0.024	0.248	0.060	0.004	0.079	<0.001
LDL-C	−0.109	<0.001	−0.118	<0.001	0.018	0.378	−0.095	<0.001	−0.005	0.821	−0.018	0.383
HDL-C	−0.104	<0.001	−0.054	0.010	0.041	0.053	−0.142	<0.001	−0.090	<0.001	−0.116	<0.001
BNP	0.038	0.102	0.081	<0.001	0.008	0.732	0.015	0.524	−0.031	0.152	−0.038	0.098
ALB	0.033	0.182	−0.072	<0.001	−0.061	0.008	0.050	0.030	0.072	0.001	0.049	0.035

### 3.3 Logistic regression analysis of the association between novel inflammatory indices and the degree of coronary artery lesions

Univariate logistic regression analysis indicated (Table 4) that MLR (OR = 4.94, 95%CI: 2.15–11.33, *P* < 0.001), NLR (OR = 1.19, 95%CI: 1.10–1.29, *P* < 0.001), and SIRI (OR = 1.41, 95%CI: 1.23–1.61, *P* < 0.001) were significantly related to severe coronary artery disease. In addition, high levels of WBC (OR = 1.13, 95%CI: 1.08–1.19, *P* < 0.001) and HbA_1_c (OR = 1.25, 95%CI: 1.16–1.35, *P* < 0.001) were associated with severe coronary artery lesions. After accounting for gender, age, BMI, hypertension, diabetes, smoking history, LVEF, HDL-C, ALB, and HbA_1_c, MLR (OR = 4.19, 95% CI: 1.46–11.98, *P* = 0.008), NLR (OR = 1.20, 95% CI: 1.09, 1.32, *P* < 0.001), and SIRI (OR = 1.31, 95% CI: 1.11–1.54, *P* < 0.001) remained notably associated with the severity of coronary artery lesions (Table 5).

**Table 4:** Univariate logistic regression analysis of the association between novel inflammatory indices and the severity of coronary artery lesions.

Variable	OR (95% CI)	*P*-value
MLR	4.94 (2.15, 11.33)	<0.001
NLR	1.19 (1.10, 1.29)	<0.001
PLR	1.00 (1.00, 1.00)	0.354
SIRI	1.41 (1.23, 1.61)	<0.001
SII	1.01 (1.01, 1.01)	<0.001
SIIRI	1.01 (1.01, 1.01)	<0.001
Age	0.99 (0.98, 0.99)	0.014
Female	0.57 (0.48, 0.68)	<0.001
BMI	1.00 (0.98, 1.03)	0.836
Diabetes	1.08 (0.92, 1.27)	0.332
Hypertension	1.05 (0.87, 1.26)	0.600
Smoke	0.94 (0.80, 1.10)	0.460
Drink	0.97 (0.82, 1.14)	0.687
NYHA III∼IV	0.84 (0.61, 1.11)	0.210
LVEF	0.00 (0.00, 0.01)	<0.001
LDL-C	1.04 (0.94, 1.15)	0.436
ALB	1.03 (1.01, 1.05)	0.030
HDL-C	0.42 (0.31, 0.57)	<0.001
WBC	1.13 (1.08, 1.19)	<0.001
HbA_1_c	1.25 (1.16, 1.35)	<0.001
HGB	1.00 (1.00, 1.01)	0.088
UA	1.00 (1.00, 1.00)	0.055

**Table 5:** Multivariable logistic regression analysis of the association between novel inflammatory indices and the severity of coronary artery lesions.

Variable	Adjusted OR (95%CI)^a^	*P*-value
MLR	4.19 (1.46, 11.98)	0.008
NLR	1.20 (1.09, 1.32)	<0.001
PLR	1.00 (1.00, 1.00)	0.057
SIRI	1.31 (1.11, 1.54)	0.001
SII	1.01 (1.01, 1.01)	0.001
SIIRI	1.01 (1.01, 1.01)	0.012

a Adjusting for gender, age, BMI, hypertension, diabetes, smoking history, LVEF, HDL-C, ALB, and HbA_1_c.

### 3.4 The predictive value of the novel inflammatory indices for the severity of coronary artery lesions

**Figure 2:**
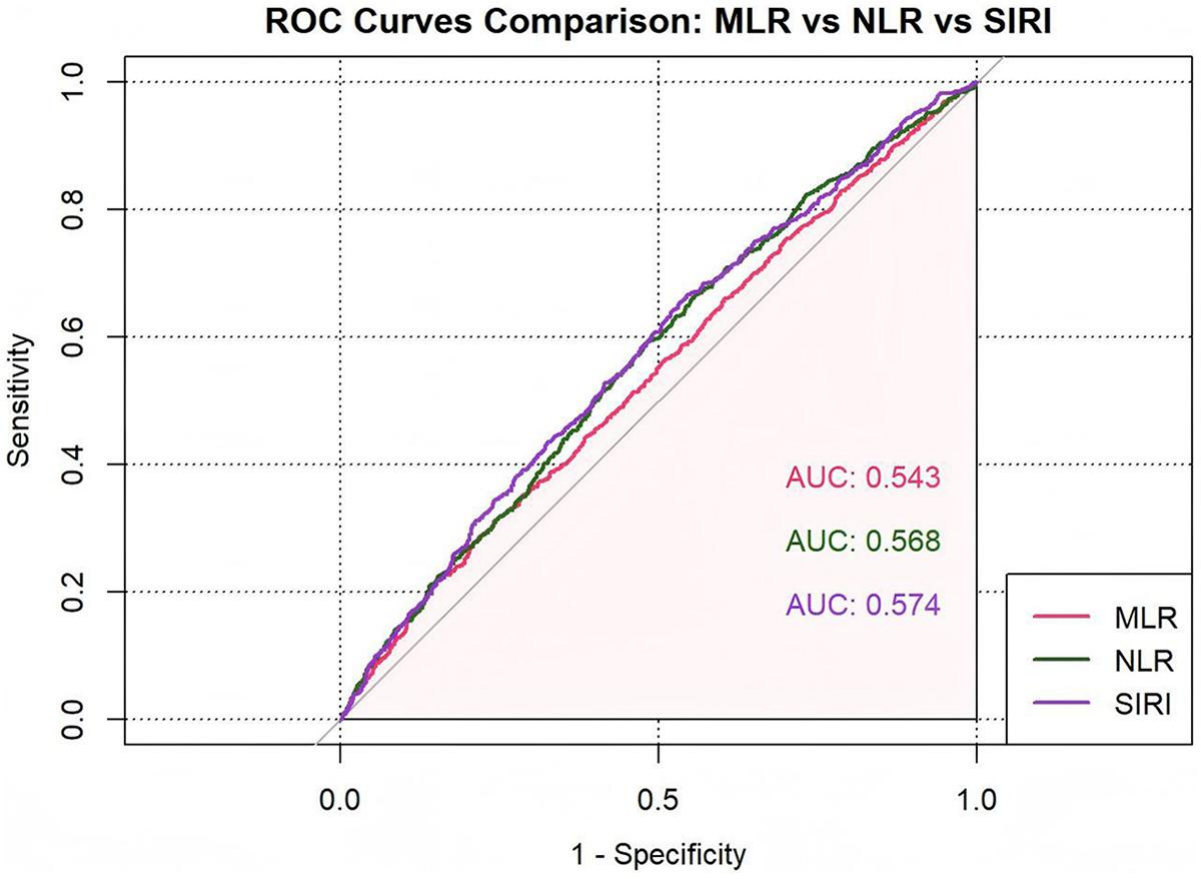
The predictive efficacy of MLR, NLR, and SIRI for the severity of coronary artery lesions.

The ROC curve was employed to evaluate the predictive value of NLR, MLR, and SIRI for the degree of coronary artery lesions in patients with SCAD (Figure 2, Table 6). The AUC values for NLR, MLR, and SIRI were 0.568 (95%CI: 0.545–0.590, *P* < 0.001), 0.543 (95%CI: 0.520–0.566, *P* < 0.001), and 0.574 (95% CI: 0.552–0.597, *P* < 0.001), respectively. Among these, NLR and SIRI showed a slight advantage over MLR in predicting the degree of coronary artery lesions.

**Table 6:** The predictive value of the novel inflammatory indices for the severity of coronary artery lesions.

Variable	AUC	95%CI	Optimal cutoff value	Specificity	Sensitivity	Z-score	*P*-value
NLR	0.568	0.545, 0.590	1.846	0.586	0.523	5.816	<0.001
MLR	0.543	0.520, 0.566	0.279	0.307	0.759	3.661	<0.001
SIRI	0.574	0.552, 0.597	0.686	0.667	0.456	6.413	<0.001

### 3.5 Exploring the linear correlation between novel inflammatory indices and the severity of coronary artery lesions through RCS curves

**Figure 3:**
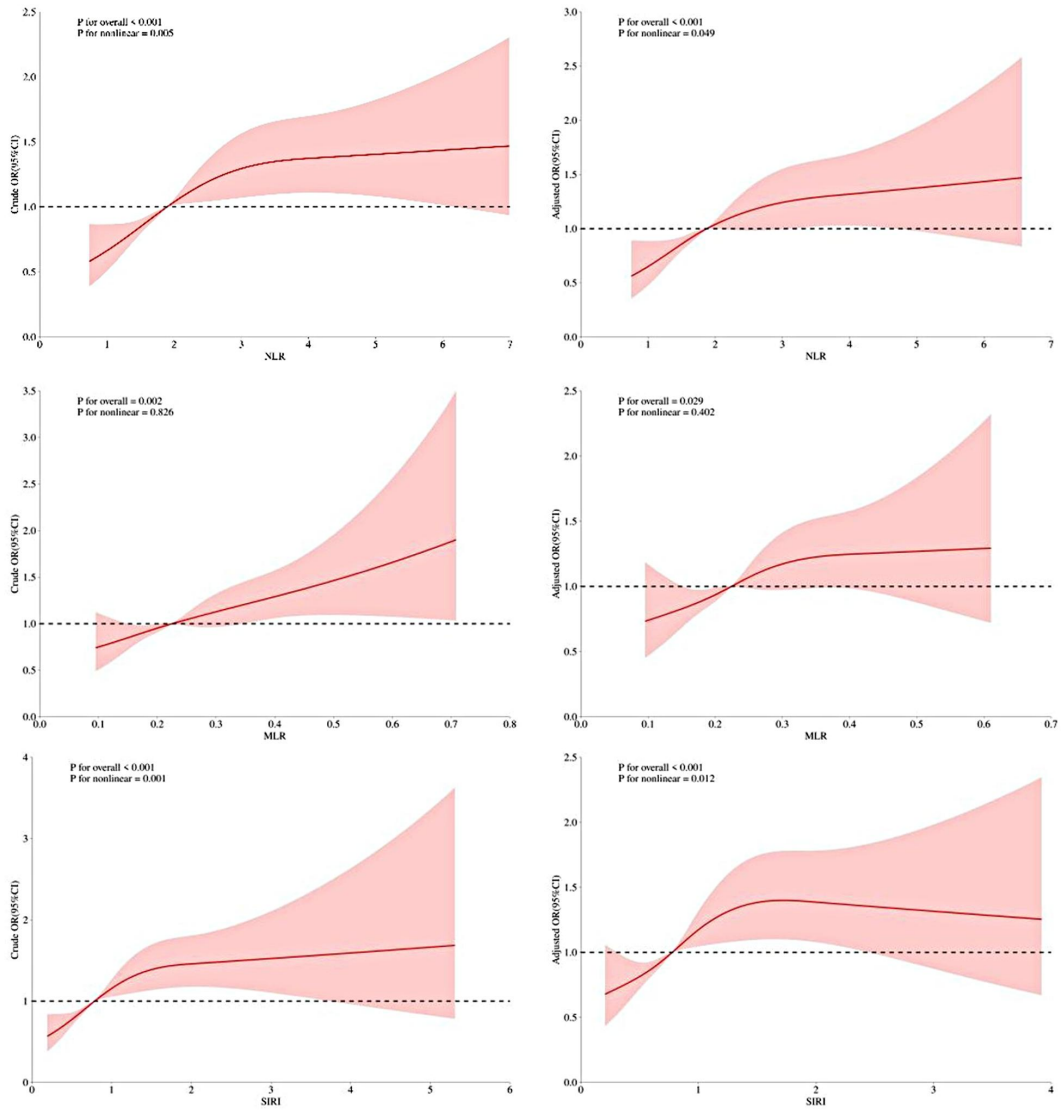
RCS curves of NLR, MLR, and SIRI and coronary artery lesion severity.

We used RCS curves to validate further the associations between NLR, MLR, and SIRI and the degree of coronary artery lesions, adjusting for gender, age, BMI, hypertension, diabetes, smoking history, LVEF, HDL-C, ALB, and HbA_1_c (Figure 3). NLR and SIRI were both closely associated with the overall severity of coronary artery lesions (*P* for overall < 0.001), and there was a nonlinear relationship (adjusted *P* for nonlinearity was 0.049 and 0.012, respectively). MLR was correlated with the overall severity of coronary artery lesions (*P* = 0.029), and no significant nonlinear relationship was found (*P* = 0.402).

## 4 Discussion

This research aimed to investigate the relationship between novel inflammatory indices and coronary lesion severity in patients with stable coronary artery disease, and to provide evidence for early risk stratification and prognostic evaluation. Through a retrospective analysis of 2424 SCAD patients, we identified the potential value of NLR, MLR, and SIRI in the assessment of coronary lesions, which was consistent with their reported roles in ACS in previous studies (4, 21). These findings implied that inflammation exerted an influence across the entire course of coronary atherosclerotic disease.

The LVEF of patients in the severe coronary artery lesions group and the negative correlation between NLR, MLR, and SIRI and LVEF are consistent with previous molecular studies that inflammation may be involved in the deterioration of cardiac function by damaging cardiomyocytes or promoting myocardial fibrosis through infiltration of immune cells, activation of inflammatory signaling pathways, and programmed cell death (22, 23). In addition, HDL-C exhibited a negative association with most of the inflammatory indices. Decreased HDL-C is linked to an increase in inflammatory activation. Specifically, decreased HDL-C leads to the stimulation of the NF-*κ*B pathway. This process is usually accompanied by an increase in interleukin-6 (IL-6), tumor necrosis factor *α* (TNF-α), and other pro-inflammatory cytokines, which contribute to inflammation via multiple mechanisms that contribute to the persistence and exacerbation of inflammation. Meanwhile, in the inflammatory state, these inflammatory factors can affect the synthesis, metabolism, and clearance of HDL-C by the liver, ultimately resulting in a decrease of HDL-C in the blood (24) (25). Remarkably, unlike ACS, PLR was not markedly different between the two groups, which may be linked to the relatively lower platelet activation in SCAD patients (5, 7); and additional verification in larger sample sizes is required. In addition, composite indices (e.g., SIIRI), although integrating more cellular components, did not correlate significantly better with Gensini scores than a single metric, suggesting that the complexity of the inflammatory network may require the combination of other indicators (e.g., cytokines or imaging parameters, etc.) to improve predictive accuracy. Restriction cubic spline (RCS) revealed a nonlinear association between NLR and SIRI and coronary lesion severity, indicating that there may be a threshold effect or a complex interplay mechanism between inflammatory intensity and lesion progression. In contrast, MLR was linearly associated with lesion severity, with no significant nonlinearity found, possibly reflecting the ongoing driving role of MLR in SCAD.

The pathogenesis of coronary atherosclerosis is mediated by intricate crosstalk between innate and adaptive immunity. Inflammasome activation facilitates the maturation and secretion of pro-inflammatory cytokines, including IL-1β and IL-18, and T lymphocyte subsets, especially regulatory T cells (Tregs), play a regulatory role in the progression and stability of atherosclerotic plaques (26). Monocyte-macrophages play an essential role in atherosclerotic plaque formation and instability. These cells not only reduce local inflammation by phagocytosis of bacteria and cellular debris but also secrete cytokines and chemokines to exacerbate the inflammatory response, which further recruit other immune cells to reach the focal area (27). The formation of extracellular traps by neutrophils can induce activation of endothelial cells, platelets, and antigen-presenting cells, leading to a pro-inflammatory immune reaction (28). Lymphocytes play a critical immunomodulatory role, and decreased lymphocyte counts are closely associated with adverse cardiovascular events (26, 29, 30). ACS triggers the activation of the hypothalamic-pituitary-adrenal axis, thereby increasing glucocorticoid secretion. Elevated glucocorticoid levels in circulation further induce migration of lymphocytes from blood to the bone marrow, causing a reduction in peripheral lymphocyte counts (29). Novel inflammatory indices can reflect the imbalance between pro-inflammatory and anti-inflammatory immune cells, and objectively evaluate the immune dysregulation status in patients with SCAD. In addition, abnormal wall shear stress (WSS) is a key hemodynamic driver of coronary inflammation and plaque progression. Low and oscillatory WSS upregulates adhesion molecules and pro-inflammatory cytokines, promoting endothelial dysfunction, leukocyte recruitment, and plaque instability. Regions with disturbed WSS coincide with higher inflammatory activity, linking hemodynamic forces to the systemic inflammatory state (31, 32).

In this study, although these novel inflammatory indices reached statistical significance, their independent clinical predictive ability for coronary artery lesions is limited. Combining additional biomarkers or imaging modalities is required to improve predictive performance. Intravascular imaging techniques hold potential value for evaluating the inflammatory status of coronary plaques. Optical coherence tomography (OCT) enables *in vivo* identification of plaque characteristics, which are correlated with the proportions of immune cells such as CD4⁺CD28^null^ T cells and Tregs (26). Near-infrared spectroscopy (NIRS) can identify plaques with lipid cores and quantify them as the lipid core burden index, which has predictive value for the prognosis of patients with CAD (33). The combination of novel inflammatory indicators and intravascular imaging techniques is expected to achieve more accurate and non-invasive assessment of plaque inflammation severity and vulnerability in patients with CAD.

In addition to lipid-lowering therapy, anti-inflammatory targeting has become an important approach to reduce residual cardiovascular risk. Colchicine can reduce the risk of cardiovascular endpoint events in patients with coronary artery disease and is approved for anti-inflammatory treatment of coronary artery disease (34, 35). The CANTOS and RESCUE trials identified IL-1*β* and IL-6 as key anti-inflammatory targets for atherosclerotic cardiovascular disease (36, 37). Although the clinical translation of related drugs still faces many challenges, targeted anti-inflammatory therapy will play an important role in future precision medicine (38). This study shows that NLR, MLR, and SIRI are independently associated with the severity of coronary artery lesions. These low-cost routine blood indicators may help screen patients suitable for individualized anti-inflammatory intervention and thus hold value for clinical translation.

Although this research reveals the potential importance of the novel inflammatory indices in SCAD, there are some limitations. (1) While a large sample size contributes to the robustness of the results, the fact that the research was carried out only in a single healthcare organization may affect the generalizability of the results. (2) This study only assessed baseline inflammatory markers without covering their dynamic changes. (3) Although this study found significant correlations between NLR, MLR, and SIRI and the severity of coronary lesions, their predictive ability was relatively limited, especially as the AUC values did not reach the desired level. (4) Due to the limited availability of ankle-brachial index and vascular imaging data of participants, complete information on peripheral arterial disease (PAD) could not be obtained. As a common comorbidity that may influence systemic inflammation, PAD limits the full adjustment for inflammatory confounding factors to a certain extent in this study. (5) Given that CRP and hs-CRP were not available for most participants, we were unable to directly compare these classic inflammatory markers with the novel indices investigated in this study. Future studies may explore the combination of other biomarkers or clinical indicators to develop a more comprehensive prediction model to improve the accuracy of assessing lesion severity in patients with coronary atherosclerotic disease.

## 5 Conclusions

In summary, this study confirms that novel inflammatory indices, including NLR, MLR, and SIRI, are significantly associated with coronary artery lesion severity in SCAD patients and are independent of traditional cardiovascular risk factors. These indicators, as non-invasive biomarkers, may provide new clues for early risk assessment and the research of inflammatory mechanisms in SCAD. Future studies should focus on optimizing the use of combinations of inflammatory indices and exploring more effective biomarkers to optimize risk stratification and therapeutic strategies.

## Data Availability

The raw data supporting the conclusions of this article will be made available by the authors, without undue reservation.
